# Continued Anesthesia with Nitrous Oxid, under Definite Pressure

**Published:** 1901-08-15

**Authors:** W. A. Heckard

**Affiliations:** Indianapolis, Ind.


### CONTINUED ANESTHESIA WITH NITROUS OXID, UNDER DEFINITE PRESSURE.

BY W. A. HECKARD, D.D.S., INDIANAPOLIS, IND.

Abstract of paper read before Tri-State Dental Meeting, Indianapolis, June, 1901.

Pure nitrous oxid, when inhaled without oxygen or air, first produces sleep. Continued, it causes anesthesia, and finally asphyxia. At this latter stage it becomes dangerous. While there are some who deny that asphyxia is at last produced, the fact that oxygen will prevent the occurrence of the symptoms, would seem to prove that the condition is properly called asphyxia. By mixing a small percentage of oxygen with the nitrous oxid, cyanosis, jerky and irregular breathing, deep stertor and movements of the muscular system are eliminated. The addition of air or oxygen renders nitrous oxid anesthesia agreeable to children, anemics, debilitated persons, patients with enlarged tonsils and lung affections, those advanced in years, and those who claim to have always heretofore had unpleasant sensations from the use of nitrous oxid alone.

All plans which depend upon the nares as an avenue for the introduction of the gas, are open to serious objections. A vast number of people are mouth-breathers, due to catarrhal affections, hay fever, hypertrophied turbinates, or nasal passages filled with polyps. Those who are subject to "bad colds in the head" are frequently unable to breathe through the nose, hence that avenue is not a favorable one for the introduction of an anesthetic. If the nasal passages are so nearly closed that breathing through them is difficult, they should not be further irritated by passing a tube through them. Furthermore, it is not infrequent that the nasal passages will spasmodically close by reflex action during anesthesia. Therefore, the nose is not the ideal avenue for introducing gas.

The objection is met by using the mouth as an avenue for introduction, this method also admits of the mixing of atmosphere with the anesthetic.

Having experimented with several styles of nozzles with which to spray the gas into the mouth, I have found that the most convenient are the ones in which the nozzle and mouth prop are combined. They are all so constructed that they can be taken apart and sterilized. When extracting is to be done, the hook nozzle will be found to fit any case, while for operating on sensitive dentin or for removing live pulps, the rubber cork and nozzle combination is better. It can be fitted to any mouth, no matter how irregular the teeth.

The holes through which the gas passes are cut in such a manner that the gas is thrown out in a flat, flame-like spray, similar to the butterfly flame of a gas burner.

My apparatus as at present constructed consists of a metallic tank, two gauges, a pressure regulator, some nozzles and a nose clamp. The tank or receiver is of fifteen gallons capacity. Small receivers do not afford an opportunity to secure an even supply of gas under a definite pressure. Without a definite pressure, capable of being regulated by the operator, sustained perfect anesthesia is impossible. Connected to the receiver are two one-hundred-gallon cylinders of nitrous oxid liquid gas for refilling the receiver. A pressure gauge indicates the amount of gas in the receiver. When a hundred-gallon cylinder of gas is allowed to flow into the receiver, the gauge will show a pressure of one hundred and twenty pounds to the square inch, and is now *compressed* gas instead of *liquid* gas.

Anesthetization is begun with a low pressure, which is gradually increased as the patient passes under the influence of the gas. When the patient is sufficiently anesthetized, the regulator may remain set at any pressure necessary to maintain any desired stage of anesthesia for an indefinite period, leaving the operator with both hands free to work, and the patient with the mouth open. It is desirable during some operations to have the patient but partially anesthetized; this can be satisfactorily accomplished by a turn up or down of the regulator, which increases or diminishes the volume of gas.

In this connection I may state that the rapid expansion of the gas as it flows from the cylinder into the receiver renders the gas very cold, but if the receiver is filled some time before the operation, the compressed gas will be warmed by the temperature of the room, a decided advantage over injecting cold gas into the nose and throat. Cold gas may cause inflammation of the mucous membrane lining the respiratory tract, or set up a train of symptoms leading to pneumonia. Warm gas acts more quickly than cold, and less of it is needed to produce anesthesia.

The usual preliminary precautions are taken and the patient fitted with the nozzle and prop best adapted for the work in hand. The mouth should be thoroughly cleansed with borolyptol and distilled water, or something equally efficient, by means of a Davidson spray bottle and an air pressure of from thirty to forty pounds. The nozzle and nose clamp are then adjusted and the administration of the gas begun with a pressure of two or three pounds.

The sound of the escaping gas is so slight at first and is so gradually increased as the patient passes under the influence, that it is seldom noticed. This is especially true when the mouth has been previously sprayed, as the patient has become accustomed to the sound. If it is desired to decrease the amount of atmosphere being inhaled, and to hasten anesthesia, a rubber cloth, a napkin, or even the hand may be used to partially close the buccal orifice. The amount of atmosphere admitted will be regulated by the appearance and condition of the patient. This can only be determined by experience.

If the operation is the extraction of a single tooth, the assistant may shut off the gas as soon as anesthesia is complete. If the operation is one that will require some time to complete, the desired stage of anesthesia is readily maintained by increasing or decreasing the supply of gas to meet the necessities of the occasion. Sensitive dentin may be excavated, live pulps extracted, the alveolar process trephined or other operations performed with perfect satisfaction to both patient and operator. The patient does not struggle or show distress, as is so frequently the case when the face piece is used. There are no asphyxial symptoms, and recovery is prompt and complete, with no bad after-effects.

Dr. Heckard administered nitrous oxid, by his method, to a boy about eight years of age, and a surgeon removed some adenoid growths from the posterior nares through the mouth. As a demonstration of quiet anesthesia it was not entirely satisfactory as the surgeon made his operation before the child was completely anesthetized. It was not a fair demonstration of the value of the method.—Ed.

DISCUSSION.

Dr. O. N. Heise, of Cincinnati: There are several features of this process which commend it. Doing away with the face piece is an advantage, as it gives one full view of the patient's features, and it renders possible better sterilization of the mouth props. The freedom from secondary results is also a commendable feature. The advantage of having the gas under definite pressure and controllable is of great importance. It permits of quicker and more durable anesthesia, and gives opportunity for the admixture of air or oxygen as needed. I have not used this apparatus, but have had a satisfactory experience with what are called the "Marston Terminals." These are two metallic rings adjustable in the mouth and serving not only to distribute the gas uniformly but at the same time distend the lips and act as a speculum. It permits a clear view of the operation and also allows for the admission of any desired amount of air. I have used them attached directly to the condensed gas cylinder, but they would, for reasons given by the essayist, work better with the gas under pressure.

In regard to length of time patients may be kept under anesthesia by any of these methods of mixing gas and oxygen or air will necessarily be determined by each individual case and circumstance. A dog was kept under the influence of nitrous oxid and gas for three days and made a satisfactory recovery. Ordinarily it is not likely that a man could be anesthetized for more more than an hour. Few operations, especially dental, will require more than ten or fifteen minutes at most. The danger is from shock of sudden recovery after long anesthesia. For minor operations of from two to five minutes this process is ideal.

DR. J. S. CASSIDY, of Covington, Ky.: The apparatus and method of the essayist are not only practicable but scientific. Dr. Geo. Watt told us thirty years ago that air or oxygen were necessary to prevent asphyxia when administering nitrous oxid. Nitrous oxid is not decomposed in the body to any appreciable extent, consequently carbon dioxid accumulates as there is no combination of it with nitrous oxid and it remains as a poison, and possibly as an obtundent or anesthetic. The mixing of air or oxygen with nitrous oxid in its administration prevents cyanosure as it furnishes the means for removing the carbon dioxid from the tissues.

DR. EMORY, of Newark, Ohio: It appears to me that by the use of these appliances there is likely to be a considerable accumulation of saliva and blood in the mouth which must be removed or it is swallowed by the patient and is likely to induce nausea or be ejected by a spasm of coughing. Most operations about the mouth do not require prolonged anesthesia. The administration of gas is often attended with so much confusion that operative dentists do not care to bother with it. This method does not suggest less of this bother, and will doubtless have other objectionable accompanyments.

Dr. Ebersole, of Cleveland, Ohio: The exhibition clinic was not satisfactory, because the patient was not a proper subject for such a clinic and the surgeon operated too soon, but this is the rule for surgical operations of this kind.

In comparing the after-effects of nitrous oxid with chloroform and ether we find that wounds heal more promptly after nitrous oxid anesthesia, and because of its prompter effects it is easier to maintain aseptic conditions about the mouth especially. The danger of nitrous oxid anesthesia is in operating when the patient is sensible, thus producing shock to the nervous system. If too soon there is normal reflex of an excited character. If the operation is made during recovery from a prolonged anesthesia there is the same shock, but diminished recuperative power. Hence the dangers are from insufficient anesthesia and excitement or prolonged and profound anesthesia with exhaustion.

Dr. C. R. Butler, of Cleveland: Would prefer to use chloroform for children, as it is more quieting and there is less hemorrhage.

Dr. Douglass, of Romeo, Mich.: Nitrous oxid is so safe that after forty-seven years' experience with it I do not hesitate to give it to people having diseased hearts or lungs, or to such as suffer from nervous disorders.

In fact, in proportion as there are diseased conditions, especially of the heart and nervous system, there is a positive demand for some safe and sufficient anesthetic. Have given gas to people known to be subjects of heart disease and have never had any trouble.

Dr. F. E. Logan, of Chicago: Nitrous oxid is not decomposed in the body. It is simply dissolved in the blood, and in its molecular capacity takes the place of oxygen. Chloroform and ether act upon the nerve centers and other tissues largely in their compound form. Nitrous oxid is an inert gas at body temperatures, but evidently has a chemico-physiological action which results in a contraction of the brain sheaths, producing anemia, sleep and anesthesia. When given with oxygen it produces safe and quiet anesthesia.

